# Editorial: Immunotherapy and multimodality therapy for lung cancer

**DOI:** 10.3389/fimmu.2024.1372513

**Published:** 2024-01-26

**Authors:** Chenghu Song, Weici Liu, Yuan Wan, Wenjun Mao

**Affiliations:** ^1^ Department of Thoracic Surgery, The Affiliated Wuxi People’s Hospital of Nanjing Medical University, Wuxi People’s Hospital, Wuxi Medical Center, Nanjing Medical University, Wuxi, Jiangsu, China; ^2^ The Pq Laboratory of BiomeDx/Rx, Department of Biomedical Engineering, Binghamton University, Binghamton, NY, United States

**Keywords:** lung cancer, immunotherapy, immune checkpoint inhibitors, multimodality therapy, predictive biomarker

Lung cancer stands as one of the most prevalent malignant tumor worldwide, with non-small cell lung cancer (NSCLC) accounting for approximately 85% of all lung cancer cases (1). The undesirable prognosis of NSCLC, with a mere 17.8% five-year survival rate due to the advanced-stage diagnosis, greatly poses challenges to modern medicine. Lung cancer treatment incorporates a series of diverse therapeutic approaches, ranging from traditional surgical resection, chemoradiotherapy to emerging targeted therapy and immunotherapy (2). The advances of lung cancer therapeutics have significantly improved patient prognosis.

Since the year of 2013, the therapeutic paradigm for lung cancer has undergone a remarkable shift with the rapid development of immunotherapy. Particularly, immune checkpoint inhibitors (ICIs) occupy a pivotal place in immunotherapy (3). The programmed cell death protein-1/programmed cell death ligand-1 (PD-1/PD-L1) and cytotoxic T lymphocyte antigen-4 (CTLA-4) have displayed desirable performances in terms of safety, efficacy and anti-tumor activity, greatly extending their clinical applicability and playing a crucial role in the treatment of ‘immuno-hot’ lung tumor (2). The efficacy of immunotherapy is based on the effective activation of immune cells to recognize and kill tumor cells. Gessner et al. revealed a surging in the number of absolute transitional B cells and activated cytotoxic T lymphocytes in NSCLC patients receiving immunotherapy or immunochemotherapy.

As one of the broadly applied monoclonal antibodies, Pembrolizumab nearly doubled the five-year survival rate to 31.9% for lung cancer patients in comparison to the 16.3% in the chemotherapy group (4). For advanced NSCLC patients with over 50% PD-L1 expression, pembrolizumab substantially extended both progression-free survival (PFS) and overall survival (OS) compared with conventional platinum-based chemotherapy (5). As a pivotal member in lung cancer immunotherapy, the approval of pembrolizumab by the Food and Drug Administration (FDA) signifies a new era in treating this challenging disease. Other monoclonal antibodies for lung cancer treatment mainly include cetuximab, bevacizumab and nivolumab, also having favorable clinical efficacy. In this Research Topic, Chen et al. challenged the established norms and demonstrated the potential of persistent PD-1/PD-L1 immunotherapy even after the progression of advanced lung cancer, which hints at promising survival benefits. This approach could reshape the therapeutic landscape of advanced lung cancer. Wang et al. uncovered that adjuvant immunotherapy had an advantage over conventional neoadjuvant chemotherapy and neoadjuvant targeted therapy for advanced resectable lung adenocarcinoma patients in terms of surgical outcomes, while neoadjuvant immunotherapy showcased superiority over neoadjuvant targeted therapy in lung squamous carcinoma cases. For patients with stage III NSCLC, Yang et al. reported that the induction and consolidation ICIs in combination with chemoradiotherapy showed superior efficacy and manageable toxicity. For unresectable stage III NSCLC, Guan et al. discovered that induction chemoimmunotherapy was safe and could improve chemoradiotherapy-related adverse events, even further enhancing treatment response and survival outcomes.

While immunotherapy holds promising prospects, it is crucial to recognize that nearly 70% of advanced NSCLC and 80% of SCLC patients fail to achieve long-term benefits from it alone. This dilemma underscores the requirement for further investigations into the underlying resistance mechanisms like poor immunogenicity, immunosuppressive tumor microenvironment, and T-cell exhaustion (6). Previous research has indicated the potential for enhancing the survival duration of patients undergoing single-agent immunotherapy treatment (7). Given the limited response rates, high costs and potential side effects of immunotherapy, it is urgently demanded to identify robust predictive biomarkers that can guide immunotherapy selection and to explore novel therapeutic targets or combination approaches that can enhance efficacy and minimize toxicity (8).

The PD-L1 expression remains the most established biomarker for guiding immunotherapy selection in lung cancer patients, yet it still has limitations and some novel biomarkers are needed (9). In this Research Topic, several biomarker studies preliminarily revealed the desirable predictive performances of novel markers, such as cancer-associated fibroblasts (CAFs) by Ren et al., T-cell Activation GTPase Activating Protein (TAGAP) by Xu et al., FAS-associated death structural domain (FADD) proteins by He et al., neutrophil-to-lymphocyte ratio (NLR) by Wang et al. and m6A demethylases by Yu et al. Additionally, Liang et al. delves into the current landscape and future possibilities of both individual and combined biomarkers for lung cancer immunotherapy. Concretely, it summarizes some individual markers like PD-L1, tumor mutation burden (TMB), hematological markers, and even specific gene mutations. Aside from these, it also deals with combined biological markers, including radiological and radiomic markers, as well as prediction models that effectively predict immunotherapy responses in NSCLC patients.

Despite limitations in current ICIs, some novel checkpoints have been uncovered like TIGIT, LAG-3, TIM-3, NKG2A, and CD73. These latent checkpoints, currently undergoing rigorous validation, hold great promise for overcoming resistance to conventional ICIs and potentially revolutionizing lung cancer treatment (10). As for the multimodality therapy, previous studies showed the feasibility of anti-angiogenic drugs to activate the immune system, thereby achieving the combination of lung cancer immunotherapy and synergistic inhibition of tumor activity (11). Li et al. revealed the superiority of combining immunotherapy with chemotherapy and anti-angiogenic drugs in the treatment of advanced NSCLC. This finding was corroborated by a systematic review by Chen et al. incorporating 16 studies and 931 patients, which found that ICIs in combination with anti-angiogenic drugs had good efficacy and safety in the second or later-line treatment of NSCLC. In addition, Chen et al. first revealed the synergistic anti-cancer effects of *Plasmodium* immunotherapy combined with gemcitabine, which were partially attributed to the inhibition of epithelial-mesenchymal transition of tumor cells by blocking the CXCR2/TGF-β-mediated PI3K/Akt/GSK-3β signaling pathway. Similarly, Liu et al. discovered that bevacizumab-loaded CalliSpheres bronchial artery chemoembolization in combination with immunotherapy and targeted therapy was effective and safe in patients with advanced lung adenocarcinoma.

Intriguingly, some external elements can enhance anti-tumor immunity or synergize with cancer immunotherapy beyond our expectation. Miao et al. summarized the potential of single and compound Chinese herbal medicines in regulating cellular pathways, enhancing immunotherapy responses, and inducing tumor cell apoptosis. Notably, it underscored the exciting possibilities of combining traditional Chinese medicine with the burgeoning immunotherapy. Luo et al. summarized the underlying mechanisms by which exercise exerts anti-tumor effects on lung cancer from four aspects—the tumor microenvironment, matrix regulation, apoptosis and angiogenesis, and highlighted the importance of personalized prescription. Moreover, Yang et al. conducted a innovative meta-analysis, discovering that radiofrequency ablation in combination with chemotherapy could improve the OS of NSCLC patients and reduce pulmonary metastases compared with radiofrequency ablation or chemotherapy alone.

This Research Topic delves into the important contributions of clinical scholars in revolutionizing lung cancer treatment through immunotherapy, multimodality approaches, and precision biomarker-driven prognosis. While ICIs have extensive application prospects for advanced NSCLC, their limitations in small cell lung cancer and emerging resistance to ICIs necessitate the therapeutic diversification. Clinical scholars can address these challenges through combining immunotherapy with established therapies such as chemotherapy, radiotherapy and targeted therapy. Moving forward, exploring novel therapeutic targets, combined with DNA repair targeting agents or cellular therapies, could be an exciting and challenging field that will transcend current limitations and hopefully open a new chapter in the treatment of advanced lung cancer in the near future.

## Author contributions

CS: Writing – original draft, Writing – review & editing. WL: Writing – original draft, Writing – review & editing. YW: Writing – review & editing, Conceptualization, Methodology, Project administration, Supervision. WM: Writing – review & editing, Conceptualization, Funding acquisition, Methodology, Project administration, Supervision.
